# Ageing with HIV: a longitudinal study of markers of resilience in young adults with perinatal exposure to HIV, with or without perinatally acquired HIV

**DOI:** 10.1002/jia2.25982

**Published:** 2022-09-29

**Authors:** Patricia A. Sirois, Yanling Huo, Molly L. Nozyce, Patricia A. Garvie, Lynnette L. Harris, Kathleen Malee, Robin McEvoy, Claude A. Mellins, Sharon L. Nichols, Renee Smith, Katherine Tassiopoulos, for the Pediatric HIV/AIDS Cohort Study

**Affiliations:** ^1^ Department of Pediatrics Tulane University School of Medicine New Orleans Louisiana USA; ^2^ Center for Biostatistics in AIDS Research Harvard T.H. Chan School of Public Health Boston Massachusetts USA; ^3^ Department of Pediatrics Jacobi Medical Center Albert Einstein College of Medicine Bronx New York USA; ^4^ Research Department Children's Diagnostic & Treatment Center Fort Lauderdale Florida USA; ^5^ Department of Pediatrics Baylor College of Medicine Houston Texas USA; ^6^ Departments of Infectious Diseases and Psychiatry and Behavioral Science Northwestern University Feinberg School of Medicine Chicago Illinois USA; ^7^ Department of Pediatrics, Infectious Diseases University of Colorado School of Medicine Children's Hospital Colorado Aurora Colorado USA; ^8^ HIV Center for Clinical and Behavioral Studies New York State Psychiatric Institute and Departments of Psychiatry and Sociomedical Sciences Columbia University New York City New York USA; ^9^ Department of Neurosciences University of California, San Diego La Jolla California USA; ^10^ Department of Pediatrics University of Illinois at Chicago Chicago Illinois USA; ^11^ Department of Epidemiology Harvard T.H. Chan School of Public Health Boston Massachusetts USA

**Keywords:** resilience, young adults, perinatal HIV‐exposed uninfected, perinatal HIV infection, milestones, lifespan development

## Abstract

**Introduction:**

Medical challenges, including perinatally acquired HIV (PHIV), can be considered adversity with the potential to compromise individuals’ ability to meet societal expectations across the lifespan. Studies suggest that resilience, defined as positive adaptation in the context of adversity, helps individuals overcome challenges and improve their quality of life. Few longitudinal studies have examined resilience in young adults with perinatally acquired HIV (YAPHIV) or perinatal HIV exposure, uninfected (YAPHEU). We examined three young adult milestones, which can affect the life‐long quality of life, as markers of resilience: high school graduation, postsecondary education and current employment.

**Methods:**

Analyses included YAPHIV and YAPHEU, ages 19–27 years, followed in longitudinal cohort studies: Pediatric HIV/AIDS Cohort Study Adolescent Master Protocol (AMP) (7–17 years) and AMP Up (≥18 years). Factors known to influence the attainment of milestones (outcomes) were examined: executive function, cognitive efficiency (working memory and processing speed), behavioural/social‐emotional functioning, parent/caregiver mental/physical health and cumulative risk. HIV disease markers for YAPHIV were examined. The most recent AMP assessment was used for each factor; outcomes were measured at AMP Up 1‐year follow‐up. Separate robust Poisson regression models were used to assess associations of each factor with each outcome; PHIV status was explored as an effect modifier of each association.

**Results:**

Participants (*N* = 315; YAPHIV = 228): 58% female, 67% Black and 27% Hispanic. Compared to YAPHEU, YAPHIV were older and from families with higher median income and fewer symptoms of parent/caregiver mental health/substance use disorders. Proportions of YAPHIV and YAPHEU, respectively, who achieved each milestone were comparable: 82% versus 78% for high school graduation (*p* = 0.49), 45% versus 51% for postsecondary education (*p* = 0.35) and 48% versus 54% for current employment (*p* = 0.32). Higher cognitive efficiency was positively associated with postsecondary education and current employment. Higher executive function, age‐appropriate behavioural/social‐emotional functioning and lower cumulative risk were associated with academic milestones. Among YAPHIV, positive associations were: higher current CD4 with postsecondary education and lower nadir CD4 with current employment. PHIV status did not modify any association.

**Conclusions:**

YAPHIV and YAPHEU demonstrated resilience, attaining at least one young adult milestone. Cognitive, behavioural and social resources to support resilience in childhood and adolescence may provide the foundation for continued achievement throughout adulthood.

## 1 INTRODUCTION

Resilience, defined as positive adaptation in the context of risk or adversity [1, 2], is instrumental to the quality of life and attainment of goals in young adulthood, particularly in the areas of work and vocational or higher education. Quality‐of‐life indicators in adulthood, such as financial stability, physical and mental health, and life expectancy, are improved with the attainment of high school and postsecondary education [3]. There is little information about high school graduation and higher education in the population with perinatal HIV exposure or perinatally acquired infection in the United States. Therefore, we examined the attainment of three societal milestones known to contribute to the life‐long quality of life: high school graduation, postsecondary education and employment.

Perinatal HIV exposure, with or without perinatally acquired HIV, can confer risks to development and wellbeing in childhood [4, 5, 6, 7, 8, 9, 10] and subsequently to quality of life in adulthood. Results from the Pediatric HIV/AIDS Cohort Study (PHACS) documented lowered performance in children and adolescents with perinatal HIV exposure compared to nationally representative test standardization samples on measures of key developmental domains. These include intellectual ability and academic achievement [11, 12, 13], language [14, 15], learning, memory, executive function [16, 17, 18, 19] and adaptive behaviour [11, 18]. Children and adolescents with perinatally acquired HIV (PHIV) or with perinatal HIV exposure who are uninfected (PHEU) demonstrated higher rates of behavioural or emotional problems than their peers in the general population. These rates were not attributed solely to PHIV because children with PHEU showed similar or even higher rates of mental health problems [20, 21, 22]. Youth with PHIV or PHEU are often from vulnerable communities affected by poverty, racism and discrimination, familial stressors and health disparities that can affect development across multiple domains [7, 23, 24, 25, 26, 27]. Thus, it is critical to determine factors that might influence the attainment of young adult milestones as youth transition through the lifespan. This knowledge could form the basis for evidence‐based interventions to benefit this population.

Many factors are known to influence adaptation to adversity: relationships with parents/caregivers and other adults; friends and romantic partners; intelligence and problem‐solving skills; self‐control, emotional regulation and planning; and self‐efficacy and motivation [2]. Risks to optimal development include maternal education less than high school, parental divorce or death, single‐parent family, perceived individual and structural racism and witnessing violence [2, 28]. Family socio‐economic status (SES) is related to physical health and achievement of societal milestones, such as high school graduation, postsecondary education and sustained employment; thus, poverty and low SES, whether chronic or of recent onset, confer risks to development and are considered major stressors for children, adolescents and families [23, 29, 30, 31, 32]. Medical illnesses present additional stressors related to disease and treatment. Regardless of the source of stress, increases in the cumulative number of stressors have been associated with increases in child maladaptation [2, 25, 26, 27]; Rutter [33] found child psychiatric problems increased substantially when any combination of four or more stressors was present in the family. Despite this knowledge base, there is a paucity of information about HIV and its effect on goal attainment as youth with PHIV or PHEU age into young adulthood.

This study draws from research in the fields of resilience and paediatric HIV to examine, from a lifespan developmental perspective, the influence of youth and parent/caregiver characteristics on the attainment of young adult milestones of high school graduation, enrolment in postsecondary education and entry into the workforce. These outcomes reflect the attainment of societal expectations for older adolescents and young adults in the United States [3]. The hypotheses were: (1) compared to young adults with PHEU (YAPHEU), a lower proportion of young adults with PHIV (YAPHIV) will earn a high school diploma or graduate equivalency degree (GED), enrol in postsecondary education, or become employed; (2) regardless of PHIV status, difficulties in youth cognitive and behavioural development and parent/caregiver mental and physical health will adversely affect the attainment of milestones; (3) regardless of PHIV status, higher cumulative risk, that is a greater number of individual, familial and life event risks, will adversely affect the attainment of these milestones; and (4) YAPHIV with better immune function will be more likely to attain one or more milestones than those with poorer immune function.

## 2 METHODS

### 2.1 Participants

The PHACS Adolescent Master Protocol (AMP) and PHACS AMP Up are prospective cohort studies of children, adolescents and young adults with PHIV or PHEU followed for long‐term evaluation of cognitive, behavioural, social‐emotional and physical health outcomes. In 2006, AMP began enrolling children and adolescents, age 7–15 years, at 15 sites in the United States and Puerto Rico, following them until their 18th birthday. AMP follow‐up ended in 2021. In 2014, AMP Up began enrolment at 14 AMP sites for follow‐up of AMP participants and other eligible YAPHIV age 18 and older. All YAPHIV and YAPHEU age 19 and older, who were previously followed in AMP and had completed the 1‐year follow‐up visit in AMP Up, were eligible for this analysis. Semi‐annual and annual study visits in AMP included face‐to‐face testing with participants, interviews with participants and parents/caregivers and medical chart abstraction. Annual study visits in AMP Up include interviews with participants, web‐based surveys and medical chart abstraction. Informed consent and assent were obtained from parents/caregivers and participants at the time of enrolment into AMP and from participants at enrolment into AMP Up. Both protocols were reviewed and approved by Institutional Review Boards at all participating sites and Harvard T.H. Chan School of Public Health.

### 2.2 Milestone outcomes

Attainment of education and employment milestones was assessed by a web‐based survey administered 1 year after entry into AMP Up. High school graduation was defined as receipt of a high school diploma or GED; participants reporting enrolment in postsecondary education without reporting receipt of a high school diploma or GED were counted as high school graduates. Postsecondary education included vocational or technical schools, 2‐year associate degree or certification programmes, 4‐year college bachelor programmes and graduate education. Employment was defined as current part‐time or full‐time work.

### 2.3 Potential predictors

**Figure 1:**
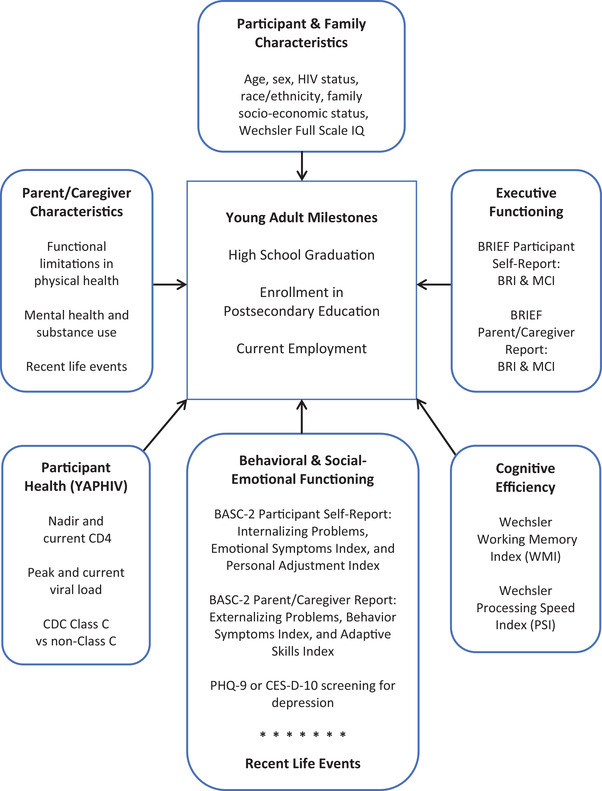
Compensatory (main effects) model of resilience in the AMP Up cohort of the Pediatric HIV/AIDS Cohort Study. Resilience was defined by participants’ attainment of one or more young adult milestones. A measure of cumulative risk developed for this study assessed the total number of risks present across all domains. All measures were collected during AMP except for the PHQ‐9 and CES‐D‐10 collected at entry into AMP Up. AMP, Adolescent Master Protocol; AMP Up, long‐term follow‐up of participants age 18 and older; BASC‐2, Behavior Assessment System for Children, Second Ed. [37]; BRIEF, Behavior Rating Inventory of Executive Function [36]; BRI, Behavioral Regulation Index; CDC, Centers for Disease Control and Prevention; CES‐D‐10, Center for Epidemiological Studies Depression Scale [42]; MCI, Metacognition Index; PHQ‐9, Patient Health Questionnaire [41]; Wechsler, Wechsler Intelligence Scale for Children, Fourth Ed. [34] or Wechsler Adult Intelligence Scale, Fourth Ed. [35]; YAPHIV, young adults with perinatally acquired HIV.

Several domains of functioning were evaluated as potentially associated with the attainment of the three outcomes (Figure 1). Measures of each domain were administered on a regular schedule during AMP follow‐up; not all measures were scheduled for the same visit. Measures selected for this analysis (Table 1) were obtained at the last AMP visit at which each measure was administered. Only those considered valid by internal test validity indices and/or examiner judgement were included. Data from AMP were used as predictors; AMP Up 1‐year follow‐up data were used for outcomes. Each measure was assessed individually to identify associations between predictors and outcomes. The measures were also combined into a study‐specific cumulative risk index, based on research indicating that cumulative stress is positively associated with adverse outcomes [2, 25, 26, 27, 33]. The index score reflected the total number of risks present across all domains. Risks were defined as follows: (1) performance greater than 1.0 standard deviation below the mean for age on the Wechsler [34, 35] Working Memory Index (WMI) and Processing Speed Index (PSI) or above cutoffs indicating clinically relevant concern on indices of the Behavior Rating Inventory of Executive Function (BRIEF) [36] and Behavior Assessment System for Children, Second Edition (BASC‐2) [37] (Table 1); (2) number of parent/caregiver‐reported symptoms of their own mental health, substance use and physical health problems >1; (3) average number of participant‐reported life events >3; and (4) number of parent/caregiver‐reported life events >3. The total score ranged from 0 to 13, determined by calculating the presence (1) or absence (0) of each risk.

**Table 1:** Potential predictors measured during AMP and included in the analysis

Domain	Measure	Selected index/subtest score
Executive functioning	Behavior Rating Inventory of Executive Function (BRIEF) [36], participant self‐report and parent/caregiver report; administered as interviews	Behavioral Regulation Index (BRI) and Metacognition Index (MCI)
Cognitive efficiency	Wechsler intelligence scales, child or adult version as appropriate for age [34, 35]; face‐to‐face testing with participant, using standardized administration procedures	Working Memory Index (WMI) and Processing Speed Index (PSI)
Behavioural and social‐emotional functioning^a^	Behavior Assessment System for Children, Second Edition (BASC‐2) [37], participant self‐report and parent/caregiver report; administered as interviews	Internalizing Problems, Emotional Symptoms and Personal Adjustment indices from the participant self‐report; Externalizing Problems, Behavioral Symptoms and Adaptive Skills indices from the parent/caregiver report
Life events	Life Events Checklist [38], participant self‐report of potentially traumatic events; administered as interview	Average life events reported during AMP follow‐up^b^
HIV disease severity (YAPHIV only)	a) CD4 count (cells/mm^3^) b) Viral load (copies/ml) c) Centers for Disease Control and Prevention (CDC) classification [39]; data obtained through medical chart abstraction	a) Nadir and most recent b) Peak and most recent c) Class C (AIDS‐defining diagnoses) versus non‐Class C
Parent/caregiver characteristics	a) Client Diagnostic Questionnaire (CDQ) [40]^c^; administered as interview b) Caregiver health interview^d^ c) Caregiver quality of life interview^d^	a) Number of positive screens for mental health and substance use disorders b) Number of functional limitations in physical health c) Number of potentially traumatic life events during 12 months prior to interview

Abbreviations: AMP, Adolescent Master Protocol; PHACS, Pediatric HIV/AIDS Cohort Study.

^a^ Depending on time of entry into AMP Up, symptoms of depression were measured with one of two depression screening instruments: the Patient Health Questionnaire (PHQ‐9) [41] or the Center for Epidemiological Studies Depression Scale (CES‐D‐10) [42]. Results are reported only for descriptive purposes. Total score ≥10 on either measure indicated a positive screen for symptoms of depression, not a diagnosis. Referrals for further clinical evaluation were provided as needed.

^b^ A participant‐reported screening measure for potentially traumatic life experiences (e.g. illness/death in family, witnessing violence and change in residence) during the 12 months prior to the interview. The average life events score (total events reported over AMP follow‐up divided by total number of interviews completed in AMP) was used in the analysis to allow examination of chronic stress rather than recent stress.

^c^ Parent/caregiver self‐report of symptoms of their own mental health and substance use. Positive scores indicated positive screens for disorders, not diagnoses.

^d^ Parent/caregiver self‐reports developed for the Pediatric AIDS Clinical Trials Group and subsequently used in PHACS.

Based upon prior research [2, 28, 29, 30, 31, 32, 33], the following participant and family characteristics were included in the multivariable models as confounding variables: participant age at the time of measurement of each potential predictor, sex, race/ethnicity, Wechsler [34, 35] Full‐Scale Intelligence Quotient (FSIQ) and family SES, using annual household income and household density.

### 2.4 Statistical analysis

Using chi‐square or Fisher's exact test, as appropriate, the proportions of participants who attained each of the three milestones were compared by PHIV status and age at outcome measurement. Each of the potential predictors, as well as proportions of participants who attained zero, one, two or all three milestones, were compared by PHIV status. Separate univariable and multivariable robust Poisson regression models were fit to evaluate the association of each measure with each milestone. Inverse probability of censoring weighting was used to adjust for potential selection bias due to loss to follow‐up (censoring) of AMP participants who did not enrol into AMP Up. Weights were generated by fitting logistic regression models for 669 AMP participants (excluding five deaths and four with no visits after enrolment into AMP), with censoring as the outcome. Variables included as predictors of censoring were age, FSIQ and education at the most recent AMP visit, sex, race/ethnicity and research site, as well as the potential predictors of the milestone outcomes. The model for YAPHIV also included CD4 and viral load at the most recent AMP visit. The weights were then incorporated into the Poisson regression models. To evaluate whether any of the associations differed by PHIV status (effect modification), an interaction term between PHIV status and each measure was added to the univariable and multivariable models.

The multiple imputation approach was used to account for missing predictor and covariate measures. Fully conditional specification with discriminant function was used to impute missing data for categorical variables; the predictive mean matching method was used to impute missing data for continuous variables. Sensitivity analyses were conducted using the complete case analysis approach, excluding participants with missing data on any measure from the multivariable models. Analyses were conducted using SAS version 9.4 (SAS Institute, Cary, NC).

## 3 RESULTS

### 3.1 Participant and parent/caregiver characteristics

AMP enrolled 678 participants. As of 1 January 2020, there were 712 participants enrolled in AMP Up, 411 of whom were previously enrolled in AMP (61%). Of the 411, 315 (228 YAPHIV and 87 YAPHEU) had completed the AMP Up Year 1 web‐based survey and were included in the analysis. Table 2 summarizes participant and parent/caregiver characteristics for YAPHIV and YAPHEU and disease severity for YAPHIV. Compared to YAPHEU, YAPHIV were older (mean age 20.2 vs. 20.8 years, respectively, *p* = 0.002; range 19–27 years), more often from families with greater financial resources per person supported (*p* < 0.001) and with parents/caregivers less likely to screen positive for mental health or substance use disorders (*p* < 0.001 and *p* = 0.03, respectively). Measures of HIV disease severity indicated that 39% of YAPHIV had nadir CD4 <200 cells/mm^3^ and 28% had received a CDC Class C classification [39] at some time in their lives. For the majority of YAPHIV, the most recent CD4 count was ≥500 cells/mm^3^ (63%), and the most recent viral load was <400 copies/ml (64%). Primary caregivers included biological parents (43% YAPHIV vs. 77% YAPHEU); biological family members (24% vs. 14%, respectively); and non‐biological family, such as adoptive and foster parents (32% vs. 9%, respectively). These data are presented for descriptive purposes and were not included in the analysis. A majority of parents/caregivers (68–72%) had a high school education or higher; 48–59% reported at least one limitation in physical health.

**Table 2:** Participant and parent/caregiver characteristics measured during AMP

		Perinatal HIV status		
		YAPHIV (*n* = 228)	YAPHEU (*n* = 87)	*p*‐value
**Participant characteristics**				
Age (years) at AMP Up Year 1 follow‐up visit	Mean (SD)	20.8 (1.6)	20.2 (1.5)	0.002^a^
Sex	M	93 (41%)	39 (45%)	0.52^b^
	F	135 (59%)	48 (55%)	
Race/Ethnicity	Black, non‐Hispanic	160 (70%)	50 (57%)	0.13^b^
	White/other, non‐Hispanic	12 (5%)	5 (6%)	
	Hispanic	55 (24%)	30 (34%)	
	Unknown	1 (0%)	2 (2%)	
Family SES	Annual income per person supported, Mdn (Q1, Q3)	$8000 ($5000, $15,000)	$5000 ($3333, $7500)	<0.001^a^
Wechsler FSIQ	Mean (SD)	84.2 (16.3)	86.3 (15.4)	0.30^a^
Nadir CD4 count, cells/mm^3^	≥500	40 (18%)	n/a	
	200–499	100 (44%)	n/a	
	<200	88 (39%)	n/a	
Most recent CD4 count,	≥500	144 (63%)	n/a	
cells/mm^3^	200–499	65 (29%)	n/a	
	<200	19 (8%)	n/a	
Peak viral load, copies/ml	≤20,000	13 (6%)	n/a	
	>20,000 to <100,000	35 (15%)	n/a	
	≥100,000	180 (79%)	n/a	
Most recent viral load,	<400	145 (64%)	n/a	
cells/mm^3^	400 to <1000	14 (6%)	n/a	
	≥1000	69 (30%)	n/a	
CDC Class C	Yes	63 (28%)	n/a	
	No	165 (72%)	n/a	
**Parent/caregiver characteristics**				
Education	High school or greater	164 (72%)	59 (68%)	0.49^b^
	Less than high school	62 (27%)	27 (31%)	
	Unknown	2 (1%)	1 (1%)	
Limitations in physical health	Yes	109 (48%)	51 (59%)	0.12^b^
	No	106 (46%)	33 (38%)	
Positive screen for mental	Yes	46 (20%)	38 (44%)	<0.001^b^
health disorder	No	150 (66%)	45 (52%)	
	Unknown	32 (14%)	4 (5%)	
Positive screen for substance	Yes	11 (5%)	11 (13%)	0.03^b^
use disorder	No	185 (81%)	72 (83%)	
	Unknown	32 (14%)	4 (5%)	
Life events in past year	0–3	168 (74%)	70 (80%)	0.63^b^
	> 3	24 (11%)	12 (14%)	
	Unknown	36 (16%)	5 (6%)	

Abbreviations: AMP, Adolescent Master Protocol; AMP Up, Adolescent Master Protocol for Participants 18 Years of Age and Older; CDC, Centers for Disease Control and Prevention [39]; n/a, not applicable; SES, socio‐economic status; Wechsler FSIQ, Wechsler Intelligence Scale for Children, Fourth Ed. [34] (ages 6–16) or Wechsler Adult Intelligence Scale, Fourth Ed. [35] (ages 17 and older) Full‐Scale Intelligence Quotient; YAPHEU, young adults with perinatal HIV exposure, uninfected; YAPHIV, young adults with perinatally acquired HIV.

^a^ *T*‐test with equal variance.

^b^ Chi‐square test.

### 3.2 Milestone outcomes

The proportions of YAPHIV and YAPHEU, respectively, who achieved each of the milestones were comparable: 82% versus 78% for high school graduation (*p* = 0.49), 45% versus 51% for postsecondary education (*p* = 0.35) and 48% versus 54% for current employment (*p* = 0.32). The proportions of YAPHIV and YAPHEU, respectively, who attained zero (11% vs. 16%), one (27% vs. 16%), two (37% vs. 37%) or all three milestones (24% vs. 31%) were also similar (*p* = 0.14). A small number of participants (*n* = 40; 13%) did not achieve any milestones (Table 3). This finding was associated with lower family SES and lower participant FSIQ, as well as lower cognitive efficiency and executive functioning (data not shown), which may be related to FSIQ. Among the 19‐year‐olds (*n* = 151), 72% had graduated high school by the AMP Up Year 1 follow‐up visit; high school graduation rates were 93% for those age 22 and older (*n* = 59) (Table 3). There was greater variability across ages in the attainment of the other two milestones.

**Table 3:** Distribution of milestones by age at AMP Up Year 1 follow‐up visit

			Age (years) at AMP Up Year 1 follow‐up visit				
Milestone		Total (*N* = 315)	19 (*n* = 151)	20 (*n* = 53)	21 (*n* = 52)	≥22 (*n* = 59)	*p*‐value^a^
High school graduation	Yes	254 (81%)	109 (72%)	43 (81%)	47 (90%)	55 (93%)	0.001
	No	61 (19%)	42 (28%)	10 (19%)	5 (10%)	4 (7%)	
Enrolment in postsecondary education	Yes	146 (46%)	67 (44%)	32 (60%)	20 (38%)	27 (46%)	0.12
	No	169 (54%)	84 (56%)	21 (40%)	32 (62%)	32 (54%)	
Current employment	Yes	156 (50%)	63 (42%)	30 (57%)	27 (52%)	36 (61%)	0.05
	No	159 (50%)	88 (58%)	23 (43%)	25 (48%)	23 (39%)	
Number of milestones attained	0	40 (13%)	29 (19%)	4 (8%)	4 (8%)	3 (5%)	0.02
	1	76 (24%)	36 (24%)	14 (26%)	13 (25%)	13 (22%)	
	2	117 (37%)	55 (36%)	14 (26%)	24 (46%)	24 (41%)	
	3	82 (26%)	31 (21%)	21 (40%)	11 (21%)	19 (32%)	

Abbreviation: AMP Up, Adolescent Master Protocol for Participants 18 Years of Age and Older.

High school graduation, high school diploma or graduate equivalency degree.

Enrolment in postsecondary education, enrolment in technical and trade schools, college (freshman to senior year), associate and bachelor degrees and graduate school.

Current employment, part‐time or full‐time employment at the time of the AMP Up Year 1 follow‐up visit.

^a^ Chi‐square test.

### 3.3 Comparisons by PHIV status

#### 3.3.1 Executive functioning

On average, YAPHIV and YAPHEU were within age expectations on the Behavioral Regulation Index (BRI) and Metacognition Index (MCI) of the BRIEF, with no significant differences between the groups (Table 4).

**Table 4:** Measures of executive functioning and cognitive efficiency collected during AMP

			Perinatal HIV status		
Domain and measures			YAPHIV (*n* = 228)	YAPHEU (*n* = 87)	*p*‐value
**Executive functioning**					
BRIEF^a^ Participant Self‐Report					
Behavioral Regulation Index (BRI)	Mean (SD)		50.3 (12.1)	50.4 (11.4)	0.95^c^
	T ≥ 65:	Yes	29 (13%)	8 (9%)	0.42^d^
		No	170 (75%)	66 (76%)	
		Unknown	29 (13%)	13 (15%)	
Metacognition Index (MCI)	Mean (SD)		51.4 (11.4)	49.5 (11.4)	0.23^c^
	T ≥ 65:	Yes	29 (13%)	8 (9%)	0.42^d^
		No	170 (75%)	66 (76%)	
		Unknown	29 (13%)	13 (15%)	
BRIEF Parent/Caregiver Report					
Behavioral Regulation Index (BRI)	Mean (SD)		52.4 (11.6)	54.9 (12.9)	0.13^c^
	T ≥ 65:	Yes	23 (10%)	12 (14%)	0.32^d^
		No	169 (74%)	60 (69%)	
		Unknown	36 (16%)	15 (17%)	
Metacognition Index (MCI)	Mean (SD)		55.6 (12.5)	53.1 (12.1)	0.14^c^
	T ≥ 65:	Yes	46 (20%)	16 (18%)	0.77^d^
		No	146 (64%)	56 (64%)	
		Unknown	36 (16%)	15 (17%)	
**Cognitive efficiency**					
Wechsler^b^ Working Memory Index (WMI)	Mean (SD)		86.8 (15.0)	90.2 (14.6)	0.08^c^
	WMI > 115		7 (3%)	4 (5%)	0.34^d^
	WMI = 85–115		113 (50%)	53 (61%)	
	WMI = 70–84		77 (34%)	22 (25%)	
	WMI < 70		23 (10%)	8 (9%)	
Wechsler^b^ Processing Speed Index (PSI)	Mean (SD)		90.6 (16.4)	94.2 (14.7)	0.07^c^
	PSI > 115		16 (7%)	7 (8%)	0.34^d^
	PSI = 85–115		133 (58%)	61 (70%)	
	PSI = 70–84		55 (24%)	14 (16%)	
	PSI < 70		16 (7%)	5 (6%)	

Abbreviations: AMP, Adolescent Master Protocol; YAPHEU, young adults with perinatal HIV exposure, uninfected; YAPHIV, young adults with perinatally acquired HIV.

^a^ BRIEF, Behavior Rating Inventory of Executive Function [36], reported as T‐scores with Mean = 50, SD = 10. T ≥ 65 is considered clinically significant.

^b^ Wechsler, Wechsler Intelligence Scale for Children, Fourth Ed. [34] (ages 6–16) or Wechsler Adult Intelligence Scale, Fourth Ed. [35] (ages 17 and older), reported as standard scores with Mean = 100, SD = 15. WMI/PSI < 70 indicates impaired performance.

^c^ *T*‐test with equal variance.

^d^ Chi‐square test.

#### 3.3.2 Cognitive efficiency

Compared to same‐age peers in the Wechsler standardization samples, on average, YAPHIV and YAPHEU were within the Wechsler Low Average to Average range for age on the WMI and PSI and did not differ statistically from one another (Table 4). However, 9–10% of participants in both groups showed impairment in working memory (WMI <70); 6–7% showed impairment in processing speed (PSI <70).

#### 3.3.3 Behavioural and social‐emotional functioning

According to the BASC‐2 parent/caregiver reports, YAPHEU were more likely to show symptoms of externalizing behaviour problems than YAPHIV, although results for both groups, on average, were within age expectations. According to participant self‐reports, there were no differences between the groups on any BASC‐2 measures of behavioural and social‐emotional functioning (Table 5); on average, both groups were within age expectations. Participants (18%) in both groups screened positive for symptoms of depression on the Patient Health Questionnaire (PHQ‐9) [41] or Center for Epidemiological Studies Depression Scale (CES‐D‐10) [42] completed at AMP Up entry.

**Table 5:** Measures of behavioural and social‐emotional functioning collected during AMP

		Perinatal HIV status		
Measure		YAPHIV (*n* = 228)	YAPHEU (*n* = 87)	*p*‐value
**BASC‐2** ^**a**^ **Participant Self‐Report**				
Internalizing Problems	Mean (SD)	48.4 (10.3)	47.0 (10.5)	0.28^c^
Clinically significant	T ≥ 70	8 (4%)	3 (3%)	0.92^d^
At risk	T = 60–69	21 (9%)	7 (8%)	
Average	T < 60	189 (83%)	76 (87%)	
	Unknown	10 (4%)	1 (1%)	
Emotional Symptoms Index	Mean (SD)	48.4 (10.4)	47.1 (10.1)	0.30^c^
Clinically significant	T ≥ 70	10 (4%)	2 (2%)	0.46^d^
At risk	T = 60–69	16 (7%)	9 (10%)	
Average	T < 60	192 (84%)	75 (86%)	
	Unknown	10 (4%)	1 (1%)	
Personal Adjustment	Mean (SD)	50.5 (9.7)	51.2 (9.8)	0.59^c^
Clinically significant	T ≤ 30	8 (4%)	2 (2%)	0.75^d^
At risk	T = 31–40	21 (9%)	10 (11%)	
Average	T ≥ 41	189 (83%)	74 (85%)	
	Unknown	10 (4%)	1 (1%)	
**BASC‐2 Parent/Caregiver Report**				
Externalizing Problems	Mean (SD)	48.6 (10.1)	52.4 (10.5)	0.003^c^
Clinically significant	T ≥ 70	9 (4%)	9 (10%)	0.07^d^
At risk	T = 60–69	17 (7%)	9 (10%)	
Average	T < 60	194 (85%)	69 (79%)	
	Unknown	8 (4%)	0 (0%)	
Behavioral Symptoms Index	Mean (SD)	49.5 (10.7)	52.3 (10.5)	0.04^c^
Clinically significant	T ≥ 70	13 (6%)	7 (8%)	0.29^d^
At risk	T = 60–69	21 (9%)	13 (15%)	
Average	T < 60	186 (82%)	67 (77%)	
	Unknown	8 (4%)	0 (0%)	
Adaptive Skills	Mean (SD)	47.8 (11.4)	47.9 (11.1)	0.94^c^
Clinically significant	T ≤ 30	15 (7%)	7 (8%)	0.63^d^
At risk	T = 31–40	54 (24%)	17 (20%)	
Average	T ≥ 41	151 (66%)	63 (72%)	
	Unknown	8 (4%)	0 (0%)	
**PHQ‐9** ^**b**^ **or CES‐D‐10** ^**b**^, Total ≥ 10	Yes	42 (18%)	16 (18%)	0.96^d^
	No	183 (80%)	71 (82%)	
	Unknown	3 (1%)	0 (0%)	

Abbreviations: AMP, Adolescent Master Protocol; YAPHEU, young adults with perinatal HIV exposure, uninfected; YAPHIV, young adults with perinatally acquired HIV.

^a^ BASC‐2, Behavior Assessment System for Children, Second Ed. [37], reported as T‐scores, Mean = 50, SD = 10.

^b^ PHQ‐9, Patient Health Questionnaire [41]; CES‐D‐10, Center for Epidemiological Studies Depression Scale [42]. Depending on time of entry into AMP Up, symptoms of depression were measured with one of two depression screening instruments. Results are reported only for descriptive purposes. Total score ≥ 10 on either measure indicated a positive screen for symptoms of depression, not a diagnosis. Referrals for further clinical evaluation were provided as needed.

^c^ *T*‐test with equal variance.

^d^ Chi‐square test.

#### 3.3.4 Cumulative risk

The average score on the cumulative risk index (Table 6) did not differ between YAPHIV and YAPHEU (3.1 vs. 3.2, respectively, *p* = 0.81), but the frequency of individual risks in several domains was greater than expected. In executive functioning, 20% of YAPHIV demonstrated risk on BRI, and 26% demonstrated risk on MCI, according to parent/caregiver reports. In cognitive efficiency, 22–44% of participants met the criteria for the definition of risk in WMI or PSI. In behavioural and social/emotional functioning, according to parent/caregiver reports, 21–23% of YAPHEU demonstrated behavioural problems, while 28–30% of both groups demonstrated lower‐than‐expected adaptive skills. Approximately 50% of YAPHIV and YAPHEU averaged more than three potentially traumatic life events per year during the course of AMP, meeting one of the definitions of risk. Parents/caregivers of YAPHIV and YAPHEU (17% vs. 31%, respectively) reported one or more difficulties in their own mental health, physical health or substance use. Parents/caregivers in both groups (11–14%) reported three or more potentially traumatic life events occurring within the 12 months prior to the interview.

**Table 6:** Risk index: frequency of participant and parent/caregiver risks by perinatal HIV status

	Perinatal HIV status		
	YAPHIV	YAPHEU	
	(*n* = 228)	(*n* = 87)	
	Risk present (*n*, %)		
**Participant risks**			
Performance discrepant from age expectations^a^ on measures of:			
Executive functioning (BRIEF)^b^			
Participant or parent/caregiver report, BRI ≥ 65	45 (20%)	19 (22%)	
Participant or parent/caregiver report, MCI ≥ 65	60 (26%)	20 (22%)	
Cognitive efficiency (Wechsler)			
WMI < 85	100 (44%)	30 (34%)	
PSI < 85	71 (31%)	19 (22%)	
Behavioral/social‐emotional functioning (BASC‐2)			
Participant self‐report, Internalizing Problems > 60	29 (13%)	10 (11%)	
Participant self‐report, Emotional Symptoms > 60	26 (11%)	11 (13%)	
Participant self‐report, Personal Adjustment < 40	29 (13%)	12 (14%)	
Parent/caregiver report, Externalizing Problems > 60	26 (11%)	18 (21%)	
Parent/caregiver report, Behavioral Symptoms Index > 60	34 (15%)	20 (23%)	
Parent/caregiver report, Adaptive Skills Index < 40	69 (30%)	24 (28%)	
Number of participant‐reported life events > 3, averaged over all life event interviews completed in AMP	110 (48%)	47 (54%)	
**Parent/caregiver risks**			
Number of parent/caregiver mental health, substance use and physical health problems > 1^c^	38 (17%)	27 (31%)	
Number of parent/caregiver‐reported life events > 3 in 12 months prior to interview	24 (11%)	12 (14%)	
Mean Index Score^d^	3.1 (2.6)	3.2 (2.9)	*p* = 0.81^d^

Abbreviations: AMP, Adolescent Master Protocol of the Pediatric HIV/AIDS Cohort Study (PHACS); BASC‐2, Behavior Assessment System for Children, Second Ed. [37]; BRIEF, Behavior Rating Inventory of Executive Function [36]; BRI, Behavioral Regulation Index; MCI, Metacognition Index; PSI, Processing Speed Index; Wechsler, Wechsler Intelligence Scale for Children, Fourth Ed. [34] (ages 6–16) or Wechsler Adult Intelligence Scale, Fourth Ed. [35] (ages 17 and older); WMI, Working Memory Index; YAPHEU, young adults with perinatal HIV exposure, uninfected; YAPHIV, young adults with perinatally acquired HIV.

^a^ Defined as T‐scores or standard scores greater than 1.0 standard deviation from the population mean.

^b^ For the BRIEF, when the participant self‐report and parent/caregiver report were both available (*n* = 225), the report with the higher score (indicating greater difficulty) was used in the analysis.

^c^ Only parents/caregivers with available data for all three measures were included in the calculation.

^d^ Each variable was assigned a score of 0 or 1 depending on the absence (0) or presence (1) of the variable. The total score for each participant ranged from 0 to 13; higher scores indicated greater total adversity.

### 3.4 Predictors of attainment of young adult milestones

**Figure 2:**
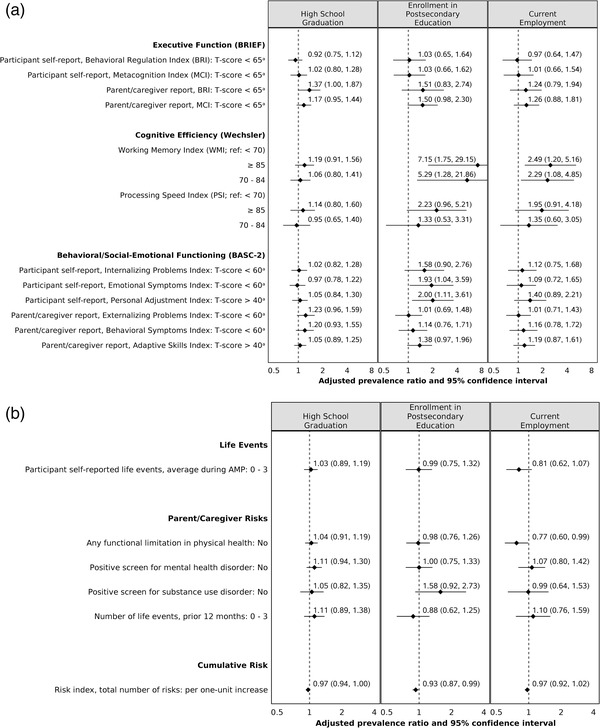
(a) Adjusted associations between predictors and attainment of young adult milestones. The association between each predictor and outcome is presented as follows: the solid diamond represents the prevalence ratio, and the horizontal line represents the 95% confidence interval. In addition, the dotted vertical line represents the null value (prevalence ratio = 1.0). The adjusted prevalence ratio for the attainment of a specific milestone compared participants with a specific predictor versus a reference group. Each model adjusted for sex, race/ethnicity, Wechsler FSIQ (except the model for cognitive efficiency due to potential overcorrection), family socio‐economic status (an index including annual income and household density) and age at the time of measurement of each predictor. ^a^Indicates lower frequency or intensity of problems. (b) Adjusted associations between predictors and attainment of young adult milestones. The association between each predictor and outcome is presented as follows: the solid diamond represents the prevalence ratio, and the horizontal line represents the 95% confidence interval. In addition, the dotted vertical line represents the null value (prevalence ratio = 1.0). The adjusted prevalence ratio for the attainment of a specific milestone compared participants with a specific predictor versus a reference group. Each model adjusted for sex, race/ethnicity, Wechsler FSIQ, family socio‐economic status (an index including annual income and household density), and age at the time of measurement of each predictor. BASC‐2, Behavior Assessment System for Children, Second Ed. [37]; BRIEF, Behavior Rating Inventory of Executive Function [36]; Risk index, a study‐specific summary of risks; Wechsler FSIQ, Wechsler Intelligence Scale for Children, Fourth Ed. [34] or Wechsler Adult Intelligence Scale, Fourth Ed. [35] Full‐Scale Intelligence Quotient.

**Figure 3:**
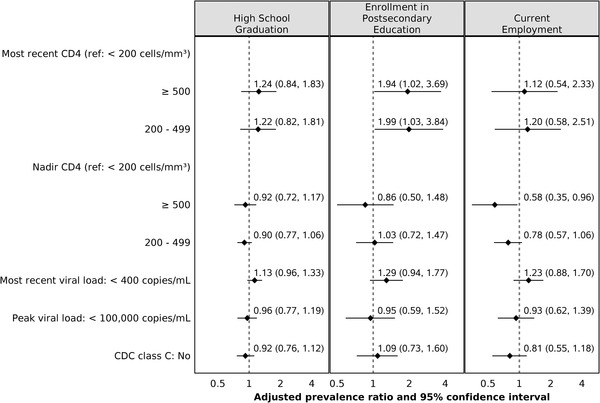
Adjusted associations between measures of HIV disease severity and attainment of young adult milestones in YAPHIV. The association between each predictor and outcome is presented as follows: the solid diamond represents the prevalence ratio, and the horizontal line represents the 95% confidence interval. In addition, the dotted vertical line represents the null value (prevalence ratio = 1.0). The adjusted prevalence ratio for the attainment of a specific milestone compared YAPHIV participants with a specific measure of HIV disease severity (predictor) versus a reference group. Each model adjusted for sex, race/ethnicity, Wechsler FSIQ (except the models for nadir CD4 and peak viral load), family socio‐economic status (an index including annual income and household density) and age at the time of measurement of each predictor. Wechsler FSIQ, Wechsler Intelligence Scale for Children, Fourth Ed. [34] or Wechsler Adult Intelligence Scale, Fourth Ed. [35] Full‐Scale Intelligence Quotient; YAPHIV, young adults with perinatally acquired HIV.

Higher cognitive efficiency was positively associated with enrolment into postsecondary education and current employment (Figure 2a). Higher executive function, per parent/caregiver report, and lower cumulative risk were associated with a greater likelihood of attaining both academic milestones (Figures 2a and b). Age‐appropriate behaviour (BASC‐2 parent/caregiver report) was positively associated with high school graduation, while age‐appropriate adaptive skills (BASC‐2 participant self‐report and parent/caregiver report) and perceived lack of difficulty in emotional functioning (BASC‐2 participant self‐report Internalizing Problems and Emotional Symptoms indices) were positively associated with enrolment in postsecondary education. Lack of functional limitations in caregiver physical health was associated with a lower likelihood of employment (Figure 2b). For YAPHIV, positive associations were: higher current CD4 with postsecondary education and lower nadir CD4 with current employment (Figure 3). PHIV status did not modify any associations. The results of the complete case analysis approach were similar to those of the multiple imputation approach for missing data.

## 4 DISCUSSION

Resilience among YAPHIV and YAPHEU participants was demonstrated by their attainment of one or more young adult milestones. Although PHIV is an important aspect of participants’ lives, it was not determinative of success in milestone attainment. Rather, success was influenced more by participants’ development during childhood and adolescence across the cognitive, behavioural and social/emotional domains examined.

Despite well‐documented early and sometimes ongoing risks for individuals affected by HIV, the proportions of young adults who attained each milestone did not differ by PHIV status, contrary to Hypothesis 1. Attainment of milestones was positively associated with higher participant executive functioning, cognitive efficiency and behavioural/social‐emotional functioning, as well as fewer parent/caregiver risks and lower cumulative risk, supporting Hypotheses 2 and 3. For YAPHIV, higher current CD4 was positively associated with postsecondary education; the current viral load was not associated with any of the outcomes. Thus, Hypothesis 4 was partially supported. The finding that lower nadir CD4 was positively associated with current employment is counterintuitive; it is possible that residual or unmeasured confounding contributed to the observed association.

The National Center for Education Statistics (NCES) [43] reports public high school graduation rates for young adults who complete high school within 4 years of entering ninth grade. In 2019, the rates were 86% for the United States general population and 80%, 82% and 89% for Black, Hispanic and White students, respectively. In our study, 81% of the total sample (*N* = 315) met the high school graduation/GED milestone, and graduation rates increased steadily from 72% for 19‐year‐olds to 93% for those age 22 and older. The comparison between NCES data and the present study is not equal because the NCES report does not reference student age, only timely graduation, and our sample of graduates included participants who attained a GED, while the NCES sample does not. While the total high school graduation rate in our sample is consistent with NCES data for Black and Hispanic students, the relatively low proportion among 19‐year‐olds (72%) indicates the presence of difficulties that may have impeded their academic progress. Developmental delays and subsequent difficulties in cognition, executive functioning and language [8, 9, 11, 12, 13, 14, 15, 16, 17, 18, 19], and possibly school absences due to medical complications, were present throughout the lives of many PHACS participants and may contribute to the slower‐than‐expected graduation rate.

Regarding mental health, 18% of 19‐year‐olds in each group screened positive for depression, comparable to a sample of 18‐ to 29‐year‐olds in the general population who completed a similar screener [44]. In addition, participants in our sample reported symptoms of depression and anxiety at a level commensurate with their peers in a national standardization sample [37].

To our knowledge, there are only two reports examining similar milestones in longitudinal studies of YAPHIV and YAPHEU. In the Bellevue pediatric cohort study (birth years 1977–1978) conducted in New York City, 57% of YAPHIV, age 19 and older, had graduated high school or earned a GED [45]; YAPHEU were not included, thus limiting comparisons with the present study. Investigators with the Child and Adolescent Self‐Awareness and Health Study (CASAH; enrolment in 2003–2008) [46] reported on the attainment of young adult milestones among YAPHIV and YAPHEU, age 18–28 years, living in New York City: 67% graduated high school or earned a GED, 19% were in college, 42% were employed; 38% were neither in school nor working. Milestone attainment was higher in AMP Up than in CASAH, but both studies found no differences in attainment between YAPHIV and YAPHEU. Important differences between the study samples might have contributed to discrepancies in results. CASAH was recruited from four sites in New York City versus 14 sites across the United States, and participants in CASAH were older than those in AMP Up at the time milestone attainment was assessed.

Attainment of academic and employment milestones is not the only way to define resilience; however, these milestones are important in United States society and predictive of success in adulthood [3]. Understanding of HIV disease and appropriate treatment has improved substantially since the early days of the epidemic, resulting in reduced morbidity and mortality. Because of these advances, our participants were likely better able to participate in social and educational activities available to their peers, possibly contributing to the attainment of young adult milestones. It is important to note that data included in this analysis were obtained prior to the COVID‐19 pandemic. Results of future studies may differ for youth who missed social, educational and psychotherapeutic opportunities or access to comprehensive medical care due to pandemic‐related restrictions in 2020–2022.

This study has several strengths, including a large number of participants, longitudinal follow‐up through childhood and into young adulthood, and the use of standardized, well‐researched measures of functioning. Some limitations were noted. The sample might not be representative of the general population of YAPHIV or YAPHEU since all participants were involved in medical follow‐up and chose to stay engaged in longitudinal research conducted at selected sites in the United States. We cannot say how youth who remained on study differed in the attainment of young adult milestones from those who opted to discontinue participation. However, results from unweighted models were largely consistent with those from models weighted to reduce selection bias, suggesting minimal bias in the association of predictors with the milestones. A comparison group of non‐HIV‐affected children was not included. In longitudinal paediatric research, several challenges make the inclusion of an appropriate comparison group particularly difficult: (1) enrolling a sufficiently large, representative sample who do not experience the medical condition(s) under study and who may not receive potential study‐related benefits and (2) minimizing attrition among participants who are in generally good health. Our cohort is a unique one, and it was important to compare the participants’ performance to that of their age‐mates in the United States general population. The standardization samples from each instrument included in the analyses are representative of United States children and youth at various ages, providing appropriate comparison groups and allowing us to determine how well our cohort of young adults was achieving the goals expected of them in United States society. Although self‐report measures can be perceived as potentially biased, the measures included in this study were psychometrically sound and contained internal validity indices to account for response patterns. The study did not include measures of social determinants of health, structural racism or various forms of discrimination; findings from such measures could further our understanding of factors influencing the attainment of young adult milestones by YAPHIV and YAPHEU in the United States.

Due to advances in HIV medicine, the current generation of young adults with PHIV is living in comparatively good health. However, the older generation of adults over 50 living with HIV are experiencing high rates of age‐related comorbidities, including heart disease, hypertension, liver and bone disease, and neurocognitive impairment; these difficulties are occurring approximately 16 years earlier than in adults without HIV [47]. As the younger generation ages, they may become increasingly vulnerable to the medical comorbidities that the older generation is experiencing; there is already evidence of a higher risk for cardiovascular disease in youth with PHIV [48]. In our study, early difficulties did not define the participants’ ultimate ability to demonstrate resilience by achieving societal expectations, and these achievements may offer personal and societal benefits that buffer young adults from the effects of future complications. These findings highlight the need for early identification of emerging difficulties and provision of ongoing culturally relevant, community‐oriented services throughout the lifespan to support further education, sustained employment, social‐emotional wellbeing and retention in medical care for younger and older adults living with HIV.

## 5 CONCLUSIONS

Future studies of resilience in youth with PHIV and PHEU should examine additional milestones typically involved in the transition to adulthood, such as sustained employment, financial independence, romantic and committed partner/marital relationships and parenthood. Our findings suggest it is important to maintain developmental surveillance and interventions, including access to medical care and age‐appropriate multidisciplinary supports, throughout the lifespan. With targeted and timely support, we can strengthen cognitive and behavioural/social‐emotional functioning to promote resilience and thereby increase rates of education, employment and medical wellbeing among YAPHIV and YAPHEU.

## COMPETING INTERESTS

The authors have no competing interests to disclose.

## AUTHORS’ CONTRIBUTIONS

PAS and MLN conceived the idea for the study and wrote the first draft of the manuscript. YH and KT designed the statistical method and analysed the data. PAS, YH, MLN, PAG, LLH, KM, RMcE, CAM, SLN, RS and KT contributed to the study design, provided critical reviews and edited the manuscript for content. All authors read and approved the final manuscript.

## FUNDING

The study was supported by the Eunice Kennedy Shriver National Institute of Child Health and Human Development with co‐funding from the National Institute on Drug Abuse, the National Institute of Allergy and Infectious Diseases, the National Institute of Mental Health, the National Institute of Neurological Disorders and Stroke, the National Institute on Deafness and Other Communication Disorders, the National Institute of Dental and Craniofacial Research, the National Cancer Institute, the National Institute on Alcohol Abuse and Alcoholism, the Office of AIDS Research and the National Heart, Lung, and Blood Institute through cooperative agreements with the Harvard T.H. Chan School of Public Health (HD052102) (Principal Investigator: George R. Seage III; Programme Director: Liz Salomon) and the Tulane University School of Medicine (HD052104) (Principal Investigator: Russell Van Dyke; Co‐Principal Investigator: Ellen Chadwick; Project Director: Patrick Davis). Data management services were provided by Frontier Science and Technology Research Foundation (PI: Suzanne Siminski), and regulatory services and logistical support were provided by Westat, Inc (PI: Julie Davidson).

## DISCLAIMER

The conclusions and opinions expressed in this article are those of the authors and do not necessarily reflect those of the National Institutes of Health or the U.S. Department of Health and Human Services.

## Data Availability

Data are available from the corresponding author upon reasonable request.
